# Trajectories of dementia-related cognitive decline in a large mental health records derived patient cohort

**DOI:** 10.1371/journal.pone.0178562

**Published:** 2017-06-07

**Authors:** Elizabeth Baker, Ehtesham Iqbal, Caroline Johnston, Matthew Broadbent, Hitesh Shetty, Robert Stewart, Robert Howard, Stephen Newhouse, Mizanur Khondoker, Richard J. B. Dobson

**Affiliations:** 1 Department of Biostatistics and Health Informatics, Institute of Psychiatry, Psychology and Neuroscience, King’s College London, London, United Kingdom; 2 National Institute for Health Research (NIHR) Biomedical Research for mental health and Dementia Unit at South London and Maudlsey NHS Foundation Trust, London, United Kingdom; 3 South London and Maudsley NHS Foundation Trust, London, United Kingdom; 4 Department of Psychological Medicine, Institute of Psychiatry, Psychology and Neuroscience, King’s College London, London, United Kingdom; 5 Division of Psychiatry, Faculty of Brain Sciences, University College London, London, United Kingdom; 6 Farr Institute of Health Informatics Research, UCL institute of Health Informatics, University College London, London, United Kingdom; 7 Department of Population Health and Primary Care, Norwich Medical School, University of East Anglia, Norwich, United Kingdom; Universidade Federal do Rio de Janeiro, BRAZIL

## Abstract

**Background:**

Modeling trajectories of decline can help describe the variability in progression of cognitive impairment in dementia. Better characterisation of these trajectories has significant implications for understanding disease progression, trial design and care planning.

**Methods:**

Patients with at least three Mini-mental State Examination (MMSE) scores recorded in the South London and Maudsley NHS Foundation Trust Electronic Health Records, UK were selected (N = 3441) to form a retrospective cohort. Trajectories of cognitive decline were identified through latent class growth analysis of longitudinal MMSE scores. Demographics, Health of Nation Outcome Scales and medications were compared across trajectories identified.

**Results:**

Four of the six trajectories showed increased rate of decline with lower baseline MMSE. Two trajectories had similar initial MMSE scores but different rates of decline. In the faster declining trajectory of the two, a higher incidence of both behavioral problems and sertraline prescription were present.

**Conclusions:**

We find suggestive evidence for association of behavioral problems and sertraline prescription with rate of decline. Further work is needed to determine whether trajectories replicate in other datasets.

## Introduction

Patients with dementia differ from one another in how quickly they deteriorate in cognition [1]. It is likely that a number of different factors contribute to this observed heterogeneity in disease progression, the extent of which is not fully understood.

A number of approaches have previously been used to study trajectories of dementia-related decline. A quadratic path of decline has been proposed in studies following subjects with either pre-clinical dementia [2] or late onset Alzheimer’s disease (AD) [3] and mixed effects models have been used to describe some of the heterogeneity through inclusion of subject-level random effects. These random effects allow for individual-level trajectories around a mean path of decline. Such models have also been used to explore risk factors for cognitive decline [4–7]. Another approach is to group subjects into fast and slow decline categories [1, 8]. However, it is possible that multiple dementia sub-populations exist with different patterns of decline [3, 9].

In latent class growth analysis, individuals are grouped together according to their pattern of change in outcome over time [10]. When applied to cognitive assessments, trajectories of decline emerge, which may be due to unrecognized sub-populations and could reflect different underlying processes of progression. Once such trajectory memberships have been defined we can describe these sub-populations and test the ability of characteristics to discriminate between trajectory classes and predict trajectory membership. Indeed, this technique has been used previously to demonstrate the association of cognitive decline with amyloid-beta and Apolipoprotein E (APOE) e4 status in healthy older adults [11].

To understand between-subject differences in cognitive decline, we need to characterise the effect of medications and comorbidities with deterioration. For example, presence of psychosis in AD, longstanding depressive symptoms, anxiety, and antidepressant and antipsychotic medications may all affect the deterioration of cognition [12, 13].

A key challenge in these studies is to identify longitudinal cohorts of sufficient size and length of follow up to reflect the path of progression for dementia patients. The South London and Maudsley NHS Foundation Trust (SLaM) has made its pseudonymised electronic health record (EHR) available for research through the Clinical Record Interactive Search (CRIS) application [14]. The records include repeat Mini-Mental State Examination scores (MMSE) available from memory assessments, as well as patient demographics, medications and comorbidities. Previously, a 6 to 12 month window of efficacy of acetyl cholinesterase inhibitors was revealed in these records, demonstrating their usefulness for research [15]. This wealth of information provides an opportunity to study heterogeneity in cognitive decline and variables contributing to decline in a real world setting.

In this study, latent class growth analysis was applied to identify trajectories of cognitive decline in routinely collected EHRs from SLaM. Characterisation of the sub-populations identified highlighted factors associated with the observed pattern of decline.

## Materials and methods

### Sample

Our retrospective patient cohort was derived from the South London and Maudsley NHS Foundation Trust (SLaM) Clinical Record Interactive Search (CRIS) tool described previously by Stewart et al[16]. The retrospective cohort was designed to make secondary use of EHR data for the study of cognitive decline. Specifically, to explore trajectories of progression in patients who were being assessed for dementia and who were likely to receive a dementia diagnosis during follow up. This was achieved by including a) subjects who had received their first referral to older adults mental health services between January 2007 and December 2014 and b) had at least three MMSE scores recorded after the first referral date and in this same period, reflecting continued assessment for dementia. Our study was therefore able to explore changes in cognition as new cases of dementia emerge.

As no selection was made based on diagnosis, primary diagnoses received at any point during the patient journey were extracted, including dementia diagnoses; (mild cognitive impairment (MCI), Alzheimer’s disease (AD), vascular dementia (VD), Lewy body dementia (LBD) and frontotemporal dementia (FTD)) and others (depression, psychosis and psychotic symptoms, behavioral disturbances, bipolar disorder and schizophrenia) were collected. MCI is included as a dementia diagnosis to identify those with some uncertainty in diagnosis but with cognitive impairment that may lead to dementia, suggesting they are in a period of transition.

Diagnoses were recorded at multiple time points throughout the patient journey and included both primary diagnoses recorded with ICD-10 code and from discussions of primary diagnoses in free text from patient health records. ICD-10 provides guidance on diagnosing dementia, including observation of cognitive decline, information on medical history of the patient and measuring brain atrophy. However, diagnosis decisions are likely to vary with clinician practice and may rely on cognitive testing and medical history alone.

Diagnosis information was used to inform whether sub-type specific trajectories were observed.

#### Data extraction

Structured field derived information included year of birth, gender, ethnicity, retirement status, cohabiting status and Health of the Nation Outcome Scales (HoNOS) item scores. MMSE scores, primary diagnoses, medications and age left school had previously been extracted from structured fields and free text using General Architecture for Text Engineering (GATE) applications, the chosen natural language processing software for use in CRIS [14]. Details on the performance of these applications have been reported previously[16].

#### Cognitive outcome

Mini-mental state examinations are widely used by clinicians to assess the level of cognitive impairment and cognitive decline in patients undergoing assessment for dementia. This scale has been validated in a number of populations[17, 18]. These scores are the only measure available to explore cognitive change in these electronic health records and are typically recorded here as total score with numerator and denominator without individual sub-scores.

During the assessment of cognitive impairment using MMSE, a series of questions are asked relating to temporal and spatial orientation, memory, attention, language and visuospatial functions. A maximum of 30 points can be achieved. Questions could be missed because of long-standing health problems such as hearing impairments, resulting in a denominator less than 30. Observations with a denominator less than 20 were excluded. For the 7% of scores with denominators less than 30 the numerator was weighted by the ratio denominator/30.

In some records two MMSE scores appeared on the same date but with different values. The majority of these scores differed by +/- one to two points. Without further information on the most accurate score and to avoid bias in selection of values, the first recorded MMSE value was selected.

In clinical practice, MMSE scores are recorded at different times for each individual and follow-up is closely related to service use and health of the individual. In our sample, MMSE observations were made during a median follow up period of 1.70 years (IQR 0.79 to 2.99 years). 41% of individuals died during follow-up, with median time to death 3 years (IQR 1.76–4.53).

#### Demographics

Demographics extracted included gender, ethnicity, cohabiting status, retirement status and age of leaving formal education. Year of birth rather than date of birth is retained within the health records as part of the record pseudonymisation process. Age at first MMSE was therefore calculated from year of birth. Cohabiting and retirement status, representing lifestyle differences, were derived from marital status and employment status. Age of leaving formal education was used as an indicator for educational achievement, as higher educational attainment is thought to reflect greater cognitive reserve against onset of decline [19].

#### Health indicators

HoNOS are used to assess a range of outcomes in older adults with mental health problems[20]. HoNOS consist of 12 items including; behavioral disturbances, non-accidental self-injury, drink or drug abuse, cognitive problems, physical health or disability, hallucinations or delusions, depressive symptoms, other mental and behavioral problems, social or supportive relationships, activities of daily living (ADL), living situation and work and leisure activities. Each item is rated according to severity of the problem as determined by the clinician; None, Minor, Mild (intervention required), Moderate or Severe. Moderate and severe categories were combined due to the small proportion of subjects rated as severe. Scores closest to the date of the first recorded MMSE score were selected as baseline HoNOS scores. No limit was imposed on the time between the first MMSE score and HoNOS score. Time to HoNOS scores is reported below.

#### Medications

Dementia medications and medications a) whose use has been suggested as repurposed agents in dementia in Appleby et al 2013 [21], and b) are prescribed within the UK mental health care setting. The complete list of medications extracted can be found in S1 Table. Episodes of medication use are defined by prescription start and stop dates. Successive medications prescription dates within 42 days are defined as single episodes to reflect periods of repeat prescriptions. Subjects were considered on medication at baseline if the medication episode was 6 months pre or post the date of the first MMSE. Any medications prescribed to less than 1% of the sample were excluded from analysis.

### Statistical analysis

#### Identifying trajectories of cognitive decline

Baseline was the time of the first received MMSE score. Latent class growth analysis (LCGA) was used to identify trajectories of decline in repeated MMSE scores[10]. Years since first MMSE score was used as the time variable. Both linear and quadratic terms were included. A quadratic term appeared sufficient to model the non-linear trend in the data and other more flexible models, including splines were not considered. LCGA was performed using the flexmix package in R [22]. Time in years was centered to allow for interpretation of both linear and quadratic terms.

Unconditional LCGA models testing the number of trajectory classes (*k*) 1–10 were compared by Bayesian Information Criteria (BIC) and bootstrapped likelihood ratio test (BLRT) [23]. Best fitting models are reflected in low relative BIC and significant improvements in *k* over *k*-1 trajectory models as indicated by the BLRT p value < 0.05. Each subject was assigned to the class with the maximum posterior probability for subsequent analysis. Relative entropy (entropy information criterion (EIC))[24] was reported indicating class separation for each trajectory model.

#### Descriptive analysis

Global associations between baseline variables and trajectory classes were investigated using Kruskal-Wallis chi-square tests (K-W *χ*^*2*^) and Chi-square tests (*χ*^*2*^*)* (or Fisher’s exact for low frequencies). Multivariable multinomial logistic regression analysis was performed to describe the relationship between trajectory membership and baseline characteristics. A final model was chosen based on stepwise selection of variables showing statistical significance in the above tests using AIC. Our association testing occurs within a single model but the number of covariates and trajectories means a large number of comparisons were made (>100), increasing our type I error rate [25]. False Discovery Rate was used to adjust p-values [26] and the quantile for computing confidence intervals was adjusted by the proportion of significant observations to give adjusted confidence intervals[27].

#### Baseline prediction model

Support Vector Machine (SVM) Models were developed to investigate baseline characteristics that could predict trajectory membership for trajectories that were not confounded by disease stage. SVMs with Linear, Polynomial and Radial Basis Kernel Functions were tested within the R caret package [28]. Data was split into 80%: 20% training to test set. Class imbalance can lead to a learning bias towards the majority class. The training data was sampled to give an equal proportion of individuals from each trajectory. The test data was not modified to reflect prediction in the real world setting. To avoid undue influence of predictors with large numeric ranges, all categorical variables were split into binary variables and continuous variables were centered and scaled. For the HoNOS items, the rating of “none” was used as the reference category. Recursive feature elimination (RFE) using 10-fold cross-validation on the training data was repeated 5 times to select the optimal subset of predictors. Near-zero variance predictors were removed prior to RFE whilst variables were centered and scaled during RFE. Parameters for linear, polynomial and radial-basis kernel functions were tuned by 10-fold cross-validation during RFE and can be found in S2 Table. Parameter tuning was performed on the whole training data with predictors selected in RFE using 10-fold cross validation, repeated 5 times. The model with the highest accuracy was selected. Sensitivity, Specificity and Receiver Operator Curve—Area Under the Curve (AUC) were calculated to assess model performance on the test set.

#### Diagnosis across trajectories

We compared proportions of diagnoses received during follow up in each trajectory using either *χ*^*2*^ or Fisher’s tests to compare whether different diagnoses related to trajectories observed.

## Results

### Cohort characteristics

In our patient population (n = 3441), the majority was female (gender: 62% female, 38% male) and was of white ethnicity (78% white, 15% black, 4% Asian, 2% Other). The median age at baseline was 80 years (IQR 74 to 85) and median MMSE of 22 (IQR 19 to 26) at baseline. Follow up was over a median of 1.70 years (IQR 0.79 to 2.99 years).

To evaluate how close the medication episodes and HoNOS items scores occurred with respect to our defined baseline, proportion of observations occurring before or after the first MMSE date i.e. baseline were identified. Medication episodes were included if they occurred in the period from 6 months before to 6 months after baseline. 18% of medication episodes occurred on or included baseline. 13% of medication episodes occurred between 0 and 6 months before baseline. For these 13%, medication episodes ended a mean of 34 days (SD 43 days) and median of 15 days before baseline. 69% of episodes occurred between 0 and 6 months after baseline. With the episode start date occurring a mean of 41 days (SD 51 days) and median of 15 days after baseline.

For the HoNOS item scores,42% of scores occurred 0 and 3.6 years before baseline,. The mean time in days from HoNOS score to baseline was 14 days (SD 49) and a median of 5 days. For the 58% of scores occurring after between 0 and 4 years from baseline; the scores occur a mean of 43 days (SD 129 days) from baseline with a median of 1 day.

### Trajectories of decline

**Fig 1:**
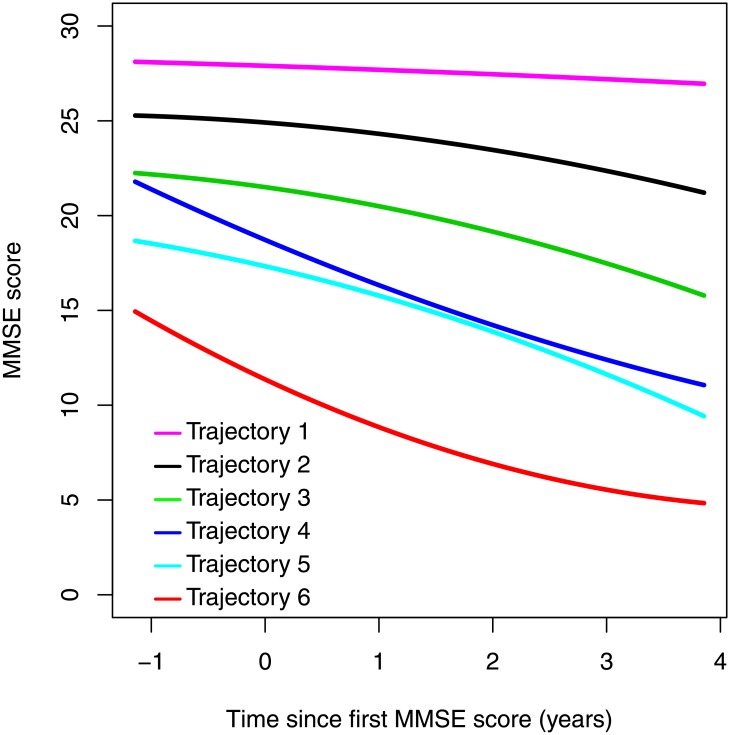
Six trajectories optimally explain the heterogeneity in cognitive decline in this SLaM NHS Trust patient sample with at least three MMSE scores. The trajectories show trend of lower initial baseline MMSE score and faster rate of decline. Trajectory 4 is distinct in that is has a similar baseline MMSE score to trajectory 3 but a similar rate to that of trajectory 6.

**Fig 2:**
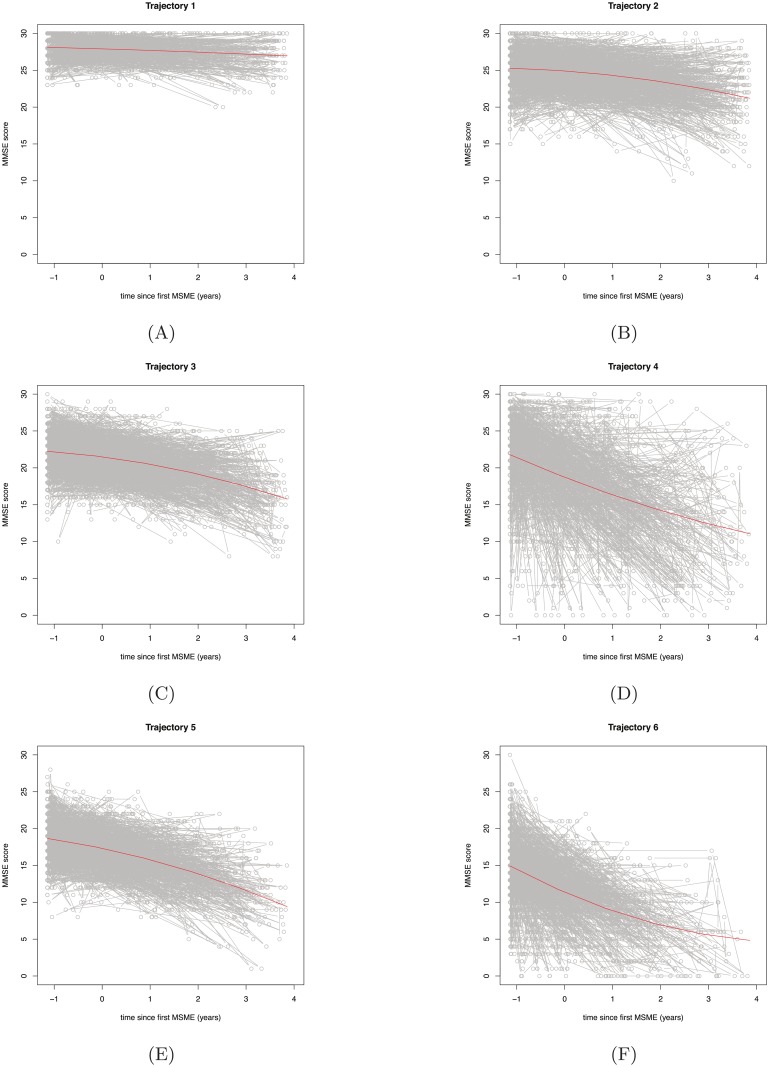
Six trajectories identified in the latent class growth analysis with estimated profile and individual level MMSE profiles. Trajectory 1 (A) to Trajectory 6 (F).

We tested how many trajectories of cognitive decline were optimal to explain the heterogeneity in the MMSE scores in this patient population. We identified the LCGA model with six trajectories as the optimal model (Table 1). The BIC was lowest for the model with nine trajectories; however, the BLRT indicated no further significant improvement in model fit after including six trajectories, so the six-trajectory model was selected (Table 1). The EIC (Table 1) suggested acceptable class separation for the model. Average and individual level MMSE profiles are presented in Figs 1 and 2 and trajectory parameters estimated in the six-trajectory model are observed in Table 2.

**Table 1:** Summary statistics from the latent class growth analysis reveals six trajectory model is optimal for explaining heterogeneity in cognitive scores.

k specified	iter	k	logLik	AIC	BIC	EIC	BLRT p value
**1**	2	1	-55682.86	111373.72	111404.78	-	-
**2**	21	2	-51276.39	102570.78	102640.67	0.85	0.1
**3**	61	3	-49669.02	99366.04	99474.76	0.84	**< 0.01**
**4**	44	4	-49002.06	98042.12	98189.67	0.79	**< 0.01**
**5**	204	5	-48702.54	97453.08	97639.45	0.76	**< 0.01**
**6**	48	6	-48466.82	96991.63	97216.83	0.75	**0.00399**
**7**	92	7	-48336.27	96740.53	97004.56	0.71	0.0549
**8**	84	7	-48336.27	96740.55	97004.57	0.71	0.323
**9**	**216**	**9**	**-48081.15**	**96250.3**	**96591.99**	**0.68**	0.123
**10**	166	9	-48114.76	96317.51	96659.2	0.68	0.540

k = number of latent classes, iterations = number of iterations before convergence, logLik = logliklihood, AIC = Akaike Information Criterion, BIC = Bayesian Information Criterion, EIC = Entropy Information Criterion, BLRT = Boostraped Likelihood Ratio Test

**Table 2:** Trajectory parameter estimates for six-trajectory latent class growth model.

Trajectory	Parameter	Estimate	SE	z value	p value
**1**	Intercept	27.9	0.0648	431	< 2.2e-16
	time (years)	-0.199	0.0446	-4.45	8.54E-06
	time^2^ (years)	-0.0122	0.0211	-0.580	0.562
**2**	Intercept	24.9	0.09051	262	< 2.2e-16
	time (years)	-0.469	0.0592	-7.92	2.37E-15
	time^2^ (years)	-0.127	0.03	-4.25	2.16E-05
**3**	Intercept	21.5	0.129	167	< 2.2e-16
	time (years)	-0.841	0.0727	-11.6	< 2.2e-16
	time^2^ (years)	-0.166	0.0376	-4.42	9.69E-05
**4**	Intercept	18.7	0.340	55	< 2.2e-16
	time (years)	-2.53	0.211	-12	< 2.2e-16
	time^2^ (years)	0.140	0.114	1.23	0.219
**5**	Intercept	17.3	0.163	106	< 2.2e-16
	time (years)	-1.37	0.113	-12.2	< 2.2e-16
	time^2^ (years)	-0.175	0.0533	-3.29	0.000985
**6**	Intercept	11.4	0.183	62.2	< 2.2e-16
	time (years)	-2.81	0.124	-22.6	< 2.2e-16
	time^2^ (years)	0.289	0.0846	3.42	0.000631

SE = standard errors, time^2^ = time squared

The majority of subjects were in trajectories 2, 3 and 5 (22%, 22% and 18%, respectively). Fewer subjects were assigned to trajectory 6 and 4 (14% and 9%), with the fastest rate of decline. 14% of subjects were assigned to the stable trajectory 1.

The trajectories identified differ in baseline MMSE score, with the lower baseline MMSE score having faster decline (Fig 1). An exception to this is trajectory 4, which had similar baseline MMSE score to trajectory 3 but a more similar rate of decline to trajectory 6.

#### Trajectory sub-population characteristics

Next we describe properties of patients within the trajectories in relation to demographics, baseline MMSE, HoNOS items and medications.

Global comparative tests highlighted differences in age (K-W χ^2^-statistic = 180.67, p-value (*p*) < 0.0001), baseline MMSE score (K-W χ^2^-statistic = 2311, *p* < 0.0001), gender (χ^2^–statistic = 21.7, degrees of freedom (df) = 5, p = 0.00059), age left school (χ^2^–statistic = 102, df = 20, *p* < 0.0001) and ethnicity (χ^2^–statistic = 31.8, df = 15, *p* = 0.00687) were found across trajectories (S3 Table).

HoNOS items that were found to be differentially represented across the trajectories included problems related to behavioral disturbance (Fisher’s exact p-value (p) = 0.0005), non-accidental self-injury (p = 0.0005), cognitive problems (p = 0.0005), physical health or disability (χ^2^–statistic = 34, df = 15, *p* = 0.00329), hallucinations or delusions (p = 0.0005), depressive symptoms (p = 0.0005), other mental or behavioral problems (χ^2^–statistic = 59.8, df = 15, *p* < 0.001), social or supportive relationships (p = 0.031), ADL (χ^2^–statistic = 340, df = 15, *p* < 0.001) and work and leisure activities (χ2 –statistic = 78, df = 15, *p*< 0.001) (S4 Table).

Differentially represented medications included donepezil (χ^2^–statistic = 121, df = 5, *p* < 0.0001), memantine (*p* = 0.0005), rivastigmine (*p* = 0.001), amlodipine (χ^2^-statistic = 12.28, df = 5, *p* = 0.031), citalopram (χ^2^-statistic = 15.2, df = 5, *p* = 0.01), fluoxetine (*p* = 0.01), sertraline (*p* = 0.001), olanzapine (p = 0.0005) and risperidone (*p* 0.0075) (S5 Table).

We performed multinomial logistic regression analysis, to identify baseline characterisitcs associated with trajectory membership. We selected trajectory 4 as the reference trajectory as firstly it does not follow a pattern of lower intercept and faster decline. Secondly, it has similar baseline MMSE score to trajectory 3 but faster decline. Comparisons to trajectory 4 may therefore highlight characteristics that may explain differences in rate of decline.

**Fig 3:**
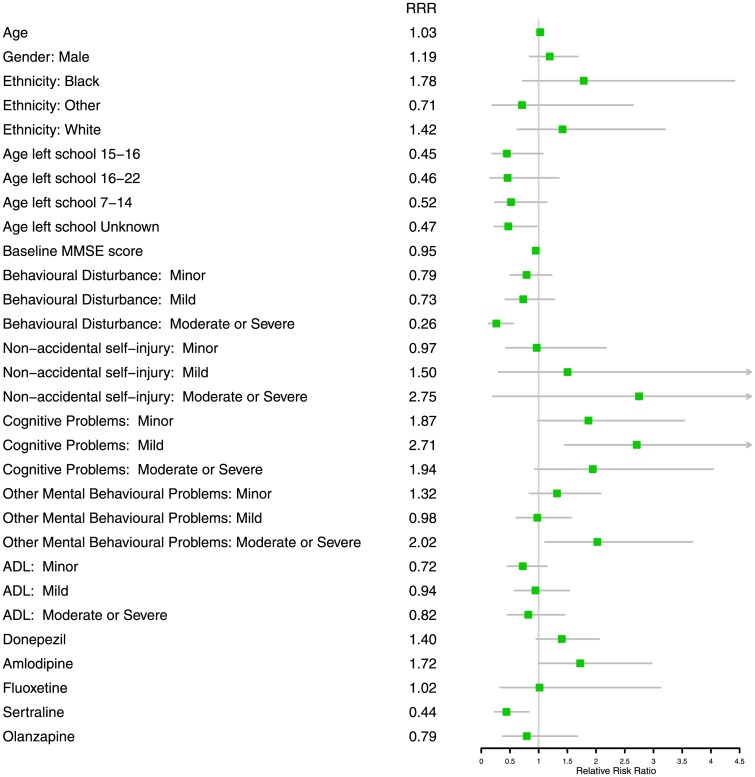
Forest plot of relative risk ratios (RRR) and confidence intervals for baseline characteristics of trajectory 3 compared to trajectory 4 from multinomial regression. Trajectory 3 is at lower risk of moderate or severe behavioral disturbances and Sertraline prescription (RRR <1), is older and at higher risk of mild cognitive problems and moderate or severe other mental health and behavioral difficulties (RRR > 1) than trajectory 4. RRR of 1 indicate no difference between trajectories. Confidence intervals including 1 are non-significant.

Adjusting for age at baseline, age left formal education, gender, ethnicity and baseline MMSE the model with lowest AIC included HoNOS items for behavioral disturbance, non-accidental self-injury, cognitive problems, other mental and behavioral problems and ADL and medications; donepezil, amlodipine, fluoxetine, sertraline and olanzapine (Fig 3, Table 3). No evidence of collinearity was observed between these covariates.

**Table 3:** Multinomial regression relative risk ratios highlight key differences in characteristics between each trajectory and trajectory four.

	1 vs 4			2 vs 4			3 vs 4			5 vs 4			6 vs 4		
	RRR	95%	CI	RRR	95%	CI	RRR	95%	CI	RRR	95%	CI	RRR	95%	CI
Age	**0.94**	**0.91**	0.98***	0.99	0.97	1.02	**1.03**	**1**	1.05*	**1.04**	**1.01**	1.07*	1.02	0.99	1.06
**Gender**															
Male	1.45	0.92	2.29	1.1	0.77	1.58	1.19	0.85	1.68	0.95	0.65	1.4	0.99	0.63	1.56
**Ethnicity**															
Black	1.08	0.35	3.35	1.13	0.46	2.73	1.78	0.72	4.41	0.95	0.36	2.56	1.69	0.49	5.83
Other	1.19	0.22	6.42	0.63	0.18	2.25	0.71	0.19	2.64	1.37	0.36	5.23	1.85	0.34	9.93
White	1.17	0.44	3.14	0.97	0.44	2.13	1.42	0.63	3.2	0.81	0.33	1.97	1.23	0.39	3.89
**Age Left School**															
15–16	0.52	0.18	1.47	0.42	0.17	1.01	0.45	0.19	1.07	0.45	0.17	1.22	0.64	0.19	2.1
16–22	0.87	0.25	2.99	0.67	0.23	1.94	0.46	0.16	1.35	0.54	0.16	1.82	0.67	0.14	3.11
7–14	0.4	0.15	1.06	**0.42**	**0.19**	0.94*	0.52	0.24	1.14	0.5	0.21	1.21	1.2	0.43	3.34
Unknown	**0.36**	**0.15**	0.86*	**0.39**	**0.19**	0.81*	0.47	0.23	0.97	0.51	0.23	1.12	0.81	0.32	2.07
Baseline MMSE score	**2.66**	**2.37**	2.99***	**1.37**	**1.28**	1.46***	0.95	0.9	1	**0.68**	**0.64**	0.72***	**0.51**	**0.47**	0.55***
**Behavioural Disturbance**															
Minor	0.56	0.3	1.06	0.72	0.45	1.15	0.79	0.51	1.23	0.76	0.47	1.24	1.04	0.6	1.81
Mild	**0.38**	**0.17**	0.84*	0.65	0.36	1.17	0.73	0.42	1.28	0.69	0.37	1.29	0.98	0.48	1.98
Moderate or Severe	**0.2**	**0.07**	0.57**	**0.29**	**0.13**	0.64**	**0.26**	**0.12**	0.56***	**0.39**	**0.18**	0.88*	0.66	0.27	1.6
**Non-accidental self-injury**															
Minor	1.84	0.68	4.93	1.03	0.45	2.34	0.97	0.43	2.17	0.98	0.39	2.44	0.47	0.15	1.45
Mild	4.12	0.72	23.72	3.7	0.79	17.22	1.5	0.3	7.58	0.97	0.14	6.51	0.34	0.03	3.63
Moderate or Severe	5.52	0.36	84.45	10.51	0.85	130.42	2.75	0.2	37.93	0.82	0.03	25.83	3.52	0.16	78.76
**Cognitive Problems**															
Minor	1.35	0.7	2.62	1.17	0.65	2.1	1.87	0.99	3.54	2.26	0.89	5.75	**10.23**	**2**	52.29**
Mild	0.78	0.38	1.57	1.61	0.9	2.87	**2.71**	**1.45**	5.06**	**4.46**	**1.83**	10.85***	**14.68**	**3.03**	71.27***
Moderate or Severe	0.65	0.23	1.82	0.91	0.43	1.92	1.94	0.93	4.04	**6.39**	**2.45**	16.68***	**28.26**	**5.59**	142.78***
**Other Mental Behavioural Problems**															
Minor	1.95	1.07	3.57	1.22	0.76	1.96	1.32	0.84	2.08	1.21	0.73	2.02	1.15	0.63	2.1
Mild	**2.21**	**1.19**	4.10*	1.38	0.86	2.23	0.98	0.61	1.56	1.35	0.81	2.24	1.28	0.71	2.31
Moderate or Severe	**3.03**	**1.44**	6.37**	1.72	0.92	3.22	**2.02**	**1.11**	3.68*	1.45	0.72	2.9	1.44	0.65	3.2
**ADL**															
Minor	**0.5**	**0.28**	0.89*	**0.5**	**0.32**	0.79**	0.72	0.46	1.14	0.79	0.47	1.34	0.96	0.5	1.86
Mild	0.71	0.37	1.34	**0.59**	**0.36**	0.97*	0.94	0.58	1.53	1.04	0.6	1.79	1.14	0.59	2.22
Moderate or Severe	**0.41**	**0.18**	0.92*	**0.43**	**0.23**	0.79**	0.82	0.46	1.45	0.73	0.38	1.38	1.29	0.61	2.71
**Anti-Dementia**															
Donepezil	0.68	0.38	1.24	1	0.67	1.51	1.4	0.96	2.05	1.08	0.71	1.65	0.97	0.59	1.58
**Antihypertensive**															
**Amlodipine**	1.1	0.54	2.26	1.71	0.98	2.99	1.72	1	2.97	1.86	1.02	3.37	**2.09**	**1.06**	4.11*
**Antidepressants**															
Fluoxetine	**4.82**	**1.15**	20.16*	1.5	0.47	4.76	1.02	0.33	3.13	0.43	0.1	1.8	0.64	0.14	2.96
Sertraline	0.7	0.31	1.56	0.51	0.27	0.98	**0.44**	**0.23**	0.83*	**0.41**	**0.19**	0.85*	**0.22**	**0.09**	0.58**
**Antipsychotics**															
Olanzapine	1.73	0.73	4.09	0.77	0.36	1.62	0.79	0.38	1.67	0.38	0.14	1.07	0.44	0.13	1.49

RRR Relative Risk Ratio from multinomial regression analysis. CI Confidence Interval, *p ≤ 0.05, **p ≤ 0.01, ***p ≤ 0.001

Relative risk ratios (RRR) for the association of covariates with membership to each trajectory can be found in Table 3. We focus on describing the differences between trajectories 3 and 4, which have different rates of decline, but very similar baseline MMSE scores estimated by the model (trajectory 3: mean = 22.2 (standard error (SE) = 0.09) and trajectory 4: mean = 21.8 (SE = 0.24)), suggesting these subjects were mild cognitively impaired at baseline[29, 30].

Subjects in trajectory 3, the slower trajectory, were slightly older than trajectory 4 (RRR = 1.03, 95% CI = 1.002–1.05) (Fig 3, Table 3). They were more likely to be rated as mild for the HoNOS cognitive problems item (RRR = 2.71, 95% CI = 1.45–5.06), the level indicating a requirement for intervention. These subjects were also less likely to have moderate/severe behavioral disturbances (RRR = 0.26, 95% CI = 0.12–0.56) and less likely have been prescribed sertraline (RRR = 0.44, 95% CI = 0.23–0.83) than trajectory 4. Trajectory 3 individuals were however, more likely to have had moderate/severe other mental health and behavioral problems (RRR = 2.02, 95% CI = 1.11–3.68) (Fig 3, Table 3).

In a sensitivity analysis, weighting each individual by class membership probability, fewer variables are selected into the model. These include HoNOS items for cognitive problems, Donepezil, Sertraline and Fluoxetine. We see comparable associations of variables with membership to trajectories 3 and 4 where included (S7 Table).

### Predicting trajectory membership

We tested the ability of baseline characteristics to predict membership to the faster declining trajectory 4, over trajectory 3.

The radial SVM model was selected, as it was a more parsimonious model, with fewer predictors than both the linear and polynomial model, but similar training accuracy. This model had an optimal sigma of 0.039 and contained 7 predictors including baseline MMSE, age, sertraline prescription and citalopram prescription. Binary variables for HoNOS items mild cognitive problems, mild physical illness or disability and mild problems with other mental health or behavioral problems were also included. On the test set, an accuracy of 0.79 (95% CI 0.73–0.85), sensitivity and specificity of 0.42 and 0.94, respectively and AUC of 0.76 was achieved. Here the accuracy is high, but misleading as with trajectory 4 as our positive class, the low sensitivity suggests a preference for classifying individuals to trajectory 3 (Table 4).

**Table 4:** Confusion matrix for classification of trajectories 3 and 4 (positive class).

	Test set class	
**Predicted class**	Four	Three
**Four**	25	9
**Three**	35	145

### Diagnosis across trajectories

**Fig 4:**
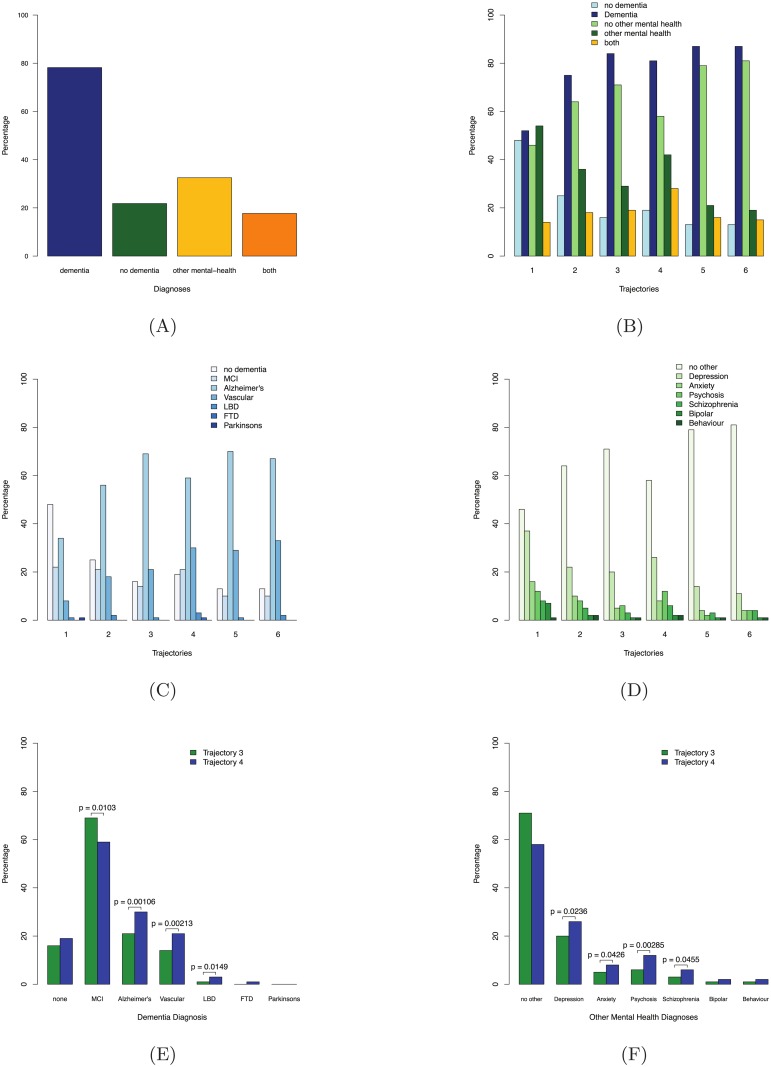
Proportions of diagnoses in the patient sample from SLaM CRIS. (A) Diagnosis types of the total sample; 78% of subjects have at least one diagnosis of dementia, 33% of subjects have at least one other mental health diagnosis, 18% have at least one of both dementia and other mental health diagnoses, (B) diagnosis types in each trajectory; Trajectory 1 has the highest proportion with other mental health diagnosis and lowest with dementia. Conversely, trajectories 5 and 6 have the highest proportion with dementia diagnoses and lowest with other mental health diagnoses. Trajectory 4 has the highest proportion of subjects with both dementia and other Mental Health diagnoses. (C) Dementia diagnoses in each trajectory; each dementia diagnosis differs significantly in proportion across trajectory classes, with the exception of FTD and Parkinson's (p < 0.05) (D) Other mental health diagnoses in each trajectory; all diagnoses differ significantly across trajectory classes with the exception of bipolar and behavioral diagnoses. (E) Comparison of dementia diagnosis in trajectories 3 and 4; significantly lower proportion of subjects with Alzheimer's diagnosis, but higher proportion of VD, MCI and LBD diagnoses in trajectory 4. (F) Comparison of other mental health diagnoses in trajectories 3 and 4; significantly higher proportion of subjects with depression, anxiety, psychosis and schizophrenia in trajectory 4.

Of those studied, 78% received one or more type of dementia diagnosis, 33% received one or more type of other mental health diagnosis and 18% of subjects received both a dementia and another mental health diagnosis (Fig 4A). MCI is included in the proportion of individuals diagnosed with dementia to reflect the uncertainty of diagnosis but suggestive evidence of path to dementia. 63.8% of individuals with MCI diagnosis receive at least one other dementia diagnosis during follow-up.

Across trajectories we saw significant differences in the proportion of both dementia diagnoses (χ2-statistic = 260, p < 0.0001) and other mental health diagnoses (χ2-statistic = 201, p < 0.0001). Trajectories 1 and 2 had the lowest proportion of subjects with dementia diagnoses (52% and 75%, respectively) but the highest with other mental health diagnoses (54% and 36%, respectively). Conversely, trajectories 5 and 6 have the highest proportion diagnosed with dementia (both 87%), particularly AD and the lowest with other mental health diagnoses (21% and 19% respectively) (Fig 4B and 4C).

The proportion of subjects with both dementia and other mental health diagnosis also differed significantly across trajectory classes (χ2-statistic = 33, p = 2.68x10^-06^). Trajectory 4 had the highest proportion of subjects diagnosed with both dementia and other mental health diagnoses (28%). It has the same rate of diagnosis of psychosis as trajectory 1 (12%) but slightly lower prevalence of depression than trajectory 1 (37% and 24%) (Fig 4B and 4D).

Taken together, these differences reflect the degree of decline in each trajectory but do not reflect trajectories of single dementia sub-types.

Comparing trajectories 3 and 4 specifically, trajectory 3 had a statistically significantly smaller proportion of subjects diagnosed with MCI (14% and 21%, p = 0.0103), VD (21% and 30%, p = 0.00213) and LBD (1% and 3%, p = 0.0149) and a higher proportion diagnosed with AD (69% and 59%, p = 0.00106) during follow up (Fig 4E). Trajectory 4 had significantly higher proportion of subjects diagnosed with depression (26% and 20%, p = 0.0236), anxiety (8% and 5%, p = 0.0426), psychosis (12% and 6%, p = 0.00285), and schizophrenia (6% and 3%, p = 0.0455) (Fig 4F).

## Discussion

Six categories of decline best explained the variability in MMSE score trajectories in 3,441 patients from a large mental health case register, who had at least 3 MMSE scores recorded during visits to dementia services between 2007 and 2014.

For the estimated trajectories, we generally found that the lower the baseline MMSE score, the faster the rate of decline observed. This pattern suggests disease stage is contributing to subject stratification for some of the trajectories. The lack of information on time since first presentation of symptoms or a secondary measure of cognition means this study is limited to account for individual differences in disease duration. This may be less of an issue in cohort studies with individuals recruited pre-symptomatically, however to our knowledge the impact of including individuals at different stages in trajectory modeling is unexplored.

Trajectories 3 and 4 had similar baseline estimated MMSE scores and are more likely to represent the consequences of different decline trajectories rather than an artifact of the known non-linear performance of the MMSE across dementia severity. Some interesting observations were made in multivariable multinomial logistic regression. A higher proportion of patients were rated as mild for the HoNOS cognitive problems item at baseline in trajectory 3.

It is evident that multiple co-morbidities were present in the sub-population in trajectory 4. Subjects were more likely to be diagnosed with depression during follow up, prescribed sertraline at baseline and more likely to have behavioral disturbances at baseline in comparison to all other trajectories. The cumulative effect of depressive symptoms is thought to contribute to increased cognitive decline [12]. Observational studies and small-scale clinical trials in Alzheimer’s subjects with depression and healthy older adults have not shown an effect of sertraline on cognitive decline [31–34], suggesting it is not the medication itself which is associated with rate of decline.

Diagnosis of psychosis and psychotic symptoms during follow up was higher in trajectory 4. Both psychosis and behavioral disturbances, agitation and aggression have been associated with a worse rate of decline in AD [35] and subjects who develop psychosis in AD may experience greater cognitive impairment[36].

The contribution of physical health co-morbidities was not explored in this study but may associate with rate of decline in dementia. Indeed, there is some evidence for an association of hypertension and cognitive decline in middle-aged individuals[37] and suggestive evidence for faster decline in subjects with both type 2 diabetes and MCI or AD diagnosis, although sample sizes for this group is small[38].

We explored whether it is possible to predict membership to the faster declining trajectory using information at baseline. Feature selection incorporates sertraline (as a possible surrogate for depression), suggested to be more likely to be prescribed in trajectory 4, mild HoNOS cognitive problems item (fewer in trajectory 4), age (trajectory 4 slightly younger than trajectory 3) and baseline MMSE, although only a small difference in mean score exists. Other selected predictors were mild other mental health or behavioral problems (less likely in trajectory 4), mild physical illness or disability and citalopram prescription, both more likely in trajectory 4. Interestingly no information on behavioral difficulties was selected, although multinomial analysis suggests subjects in trajectory 4 are more likely to have behavioral disturbances.

Whilst the model accuracy on the test set is in an acceptable range, the sensitivity is low suggesting many false negatives are possible i.e. subjects who will decline more rapidly being classified to the slower declining trajectory. Increasing the sensitivity would reduce false negatives at the cost of increasing false positives. The preference of false negative and false positive rates would depend on the clinical implications for patients being classified to the faster declining trajectory.

This difficulty in predicting membership to trajectory 4 suggests we may be missing a covariate with predictive importance. Indeed it was not possible to investigate APOE e4 status, Amyloid and Tau protein information. These variables should be explored in future studies.

Inclusion of individuals is based on 3 MMSE scores, reflecting individuals being monitored for dementia. This leaves the possibility to study individuals early in disease or who do not develop dementia during study follow up. In our analysis, these individuals are largely classified to Trajectory 1. Despite the lack of change for this trajectory, some individuals receive a dementia diagnosis during follow-up. This could be related to misdiagnosis or misclassification of individuals to trajectory 1.

Variability in diagnosis method means we do not restrict our observations to dementia diagnosis groups and are limited in our ability to study each sub-type separately due to the possibility of misdiagnosis and misclassification. Indeed, 12% of individuals have at least two different dementia sub-types diagnosed during follow-up, when excluding MCI as a sub-type. This may however, reflect the progressive nature of the disease with cognitive deficits manifesting differentially over time.

Studies exploring cognitive decline trajectories have identified between 3 and 8 trajectories[39–41], differences could be attributed to modeling with different cognitive measures and assessing decline by age instead of time. The large sample of individuals selected for this study is representative of the population being served by SLAM. Independent replication would further support these findings.

The MMSE, due to its use as a tool to explore cognitive impairment, is widely recorded in health records of older adults. Further improvements in model fit may be achieved by assuming a beta-binomial distribution for the MMSE scores[39]. Despite this and the floor and ceiling effects of the MMSE[17, 42], this study is able to detect multiple trajectories of decline in moderate-to-severe individuals. In addition, our associations of behavioral difficulties and depression between these trajectory groups are consistent with our understanding of more rapid decline previously reported.

## Conclusions

This study provides evidence for multiple trajectories of decline in dementia, using real world data from one of the largest mental healthcare providers in Europe. Despite a confounding effect of disease stage on trajectory membership, we find that differences in behavioral disturbances and antidepressant medication may be informative of rate of cognitive decline in patients with dementia.

## Supporting information

S1 Table Dementia mediations and potential repurposed agents for dementia prescribed within SLaM NHS trust selected for this analysis. (XLSX)

S2 Table Support vector machine models and tuning parameters. Values for sigma determined using sigest function from the kernlab library in R
(XLSX)

S3 Table Baseline characteristics of the six trajectories of cognitive decline. N sample size, SD standard deviation, IQR interquartile range
Omnibus p-value derived from chi-square, Fisher extact or Kruskal-Wallis tests; if Fisher extact test only p-value reported
(XLSX)

S4 Table HoNOS Item scores at baseline summarized across the six trajectories of cognitive decline. N sample size, SD standard deviation, IQR interquartile range
Omnibus p-value derived from chi-square, Fisher extact or Kruskal-Wallis tests; if Fisher extact test only p-value reported
(XLSX)

S5 Table Medications prescribed across the six trajectories at baseline. N sample size, SD standard deviation, IQR interquartile range
Omnibus p-value derived from chi-square, Fisher extact or Kruskal-Wallis tests; if Fisher extact test only p-value reported
(XLSX)

S6 Table Improvements in multinomial logistic regression model AIC when excluding named variables during stepwise selection of covariates. AIC = Akaike Information Criterion, Df = degrees of freedome, full model = model includes all variables showing differences across trajectory classes; age, gender, ethnicity, age left formal education, baseline MMSE, HoNOS items; behavioural disturbances, non-accidental self-injury, cognitive problems, physical health or disability, hallucinations and/or delusions or false beliefs, depressive symptoms, other mental health or behaviour problems, social or supportive relationships, activities of daily living (ADL) and work and leisure activities, Medications; Donepezil, Memantine, Rivastigmine, Amlodipine, Citalopram, Fluoxetine, Sertraline, Olanzapine and Risperidone.
(XLSX)

S7 Table Weighted multinomial regression relative risk ratios highlight key differences in characteristics between each trajectory and trajectory four. RRR Relative Risk Ratio from multinomial regression analysis. CI Confidence Interval, *p ≤ 0.05, **p ≤ 0.01, ***p ≤ 0.001
(XLSX)
